# Cultured Meat and Australia's Generation Z

**DOI:** 10.3389/fnut.2020.00148

**Published:** 2020-09-08

**Authors:** Diana Bogueva, Dora Marinova

**Affiliations:** ^1^Curtin University Sustainability Policy (CUSP) Institute, Curtin University, Perth, WA, Australia; ^2^Centre for Advanced Food Enginomics (CAFE), The University of Sydney, Sydney, NSW, Australia

**Keywords:** cultured meat, disgust, environmental, Gen Z, masculinity, meat alternatives, sustainability, Sydney

## Abstract

This exploratory study of Gen Z consumers (*n* = 227) examines perceptions and opinions about cultured meat of young adults residing in Sydney, Australia. It uses an online survey and describes the findings quantitatively and through the words of the study participants. The results show that the majority (72%) of the participants are not ready to accept cultured meat; nonetheless, many think that it is a viable idea because of the need to transition to more sustainable food options and improve animal welfare. When faced with a choice between different alternatives to farmed meat, a third of the participants reject cultured meat and edible insects but accept plant-based substitutes finding them more natural. Concerns about masculinity and betraying Australia as a country of quality animal meat are also raised. A significant number of young people (28%) however are prepared to try cultured meat. Environmental and health concerns may influence a broader section of society to embrace this novelty. With its power as the emerging new consumers, Gen Z is putting the future of cultured meat under scrutiny.

## Introduction

There is increasing awareness about the negative impacts of the current levels of meat consumption on the natural environment and the planet's ecosystems (1). For example, the disproportionately large appropriation of land for grazing and production of animal feed, is a major contributing factor for the highest rate of biodiversity loss and species extinction in human history (2). Further destructive consequences from excessive consumption of animal-based foods are manifested with the precarious increases in greenhouse gas emissions, freshwater use, deforestation, land, and water pollution. In 2019, there have also been significant social unrest and protests across the globe by groups concerned about climate change as well as the exploitation of animals for human consumption (3, 4). This has resulted in confrontations between those who believe that meat is an essential component of the human diet equated with good nutritional qualities, strength and masculinity (5), and those who argue that the long period of dependence on livestock as food should be assigned to the past (6). The creation of cultured meat leading to cellular agriculture is the way of resolving these tensions emerging after 20 years of scientific research and recent investment waves (7).

### Cultured Meat

Producing animal meat without livestock (8) is being described as a tissue-engineering technology which uses live cells taken painlessly from the animal's body to be proliferated and grown independently from its organism. This cultured meat, also referred to as *in-vitro*, cell-based, lab-grown, cell-cultured, fake and clean meat, is perceived to have animal welfare, environmental and health advantages over traditional meat. As cultured meat is still in its infancy, any claims about its energy or water efficiency are yet to be fully substantiated (9). An anticipatory life-cycle analysis (10) suggests that cultured meat would require smaller agricultural inputs and less land, however it would be more energy-intensive compared to livestock. Although the term meat continues to be used to indicate the animal origin of the product, it defines a new conceptual line of producing cruelty-free food. Van der Weele and Driessen (11) describe cultured meat as an ethical framework that allows people to continue to consume animal proteins while also having a meaningful relationship with farm animals.

Irrespective of the technological and production advances, a major factor for the adoption of cultured meat is its acceptance by the consumers. There is a large number of factors that shape consumer preferences ranging from sensory experiences to psychological predisposition, health considerations, environmental concerns and marketing influences (12). Age and gender also impact on people's food choices (13). In this study, we explore the attitudes of the young generation of Australians in relation to cultured meat by surveying Generation Z Sydney residents.

There have already been other studies investigating the acceptance of cultured meat in places, such as USA (14, 15) and the Netherlands (16); comparisons have been made between consumers in USA, China and India (17); the USA and the UK (18); and China, Ethiopia and the Netherlands (19). Systematic literature reviews highlight the demographic and geographic variations between consumers (20, 21) as well as the need for further research.

### Generation Z

This is the first study to explore the acceptance of cultured meat amongst Australian youth with a specific focus on Generation Z living in Sydney, Australia. Sydney is Australia's best-known iconic city which has a distinctive dynamic and multicultural atmosphere with vibrant cultural and artistic life. It is also Australia's business hub with innovation, knowledge-based industries, services, health and medical care and tourism flourishing. Sydney is consistently being ranked as one of the most liveable cities in the world characterized with economic and population growth as well as numerous opportunities supported by its modern infrastructure and competitive advantages (22). This study explores a particular section of Sydney's population, Generation Z or Gen Z—a demographic cohort following the Millennials (23) and preceding the latest Generation Alpha (24).

Generation Z (born between 1995 and 2010) represents around 20% of the current Australian population or 5 million young people (24). Its share in the world is larger at almost 30% or 2 billion people in 2020 (24). This generation grew up with digital technologies, the Internet and social media. Some argue that Gen Z is defying the reputation of entitlement characteristic for the Millennials, is “prematurely mature” [(25), p. 59] and is already exhibiting qualities, such as social generosity and environmental responsibility (26).

There is a limited number of studies specifically examining the consumer attitudes of this newly emerging world power (23, 25, 27), and particularly around their relationship with meat alternatives and cultured meat (28, 29). Although yet to be properly understood, Gen Z is not only defined by technology but is also the future economic, decision-making, political and social change driving force. They are ready to intervene and break the status quo, charged with a mission of social and environmental responsibility. Most of the knowledge, concepts and ideas this highly technologically advanced and diverse digital generation grasps from the net, exploring the vast opportunities of Google, YouTube and other social media (30). Globally aware and no strangers to social activism, the technologically empowered Gen Z wants to make a difference and to leave a mark of significance.

Gen Z has already demonstrated their strong views about the world, their own future and the need for rethinking the relationships between people and with the planet through the voices of Malala Yousafzai—the Pakistani activist for women's rights, Greta Thunberg—the Swedish environmental activist and climate change campaigner, and Billie Eilish—the American vegan singer-songwriter. This generation wants its voices and opinions to be heard, to be actively engaged in political conversations, to become influencers, to be involved and bring positive changes. They are smart, challenging, adventurous, active decision-makers (25) who are not to be underestimated with their tech-savvy skills and expertise in easily finding information. This generation is armed with learning from humanity's previous missteps, environmentally aware and tends to rally spontaneously behind global causes that resonate with them.

Despite living in a wealthy country with a prosperous economy, Australia's Gen Z is apprehensive about the world and the many environmental problems they are inheriting, with climate change being their biggest concern (31). They however believe in the power of knowledge, research, science and technology with almost half of them (49%) trusting the university sector to deliver solutions for the world's urgent and pressing challenges—a much higher share than amongst their global counterparts (31). From this point of view, it is very interesting how the Australian Gen Z in particular responds to the emerging cultured meat. However, our 2019 survey covered only adult Gen Z representatives who in that year would have been at least 18 years of age as they are already economically independent and can make their own food-related decisions. Hence, the Gen Z sample covered in this research is of Sydney residents born between 1995 and 2001. None of the participants has tasted cultured meat and their responses are based only on perceptions and the information they have had prior to the survey.

Section Materials and Methods outlines the methodology of the study before we present and discuss the results from the qualitative survey carried out in Sydney in 2019. The final section closes the discussion by reflecting on the dynamically changing circumstances that may speed up consumers' decision about cultured meat. Theoretically, the process of cultured meat production could efficiently supply enough products to satisfy the global demand for meat; the reality however will depend on the existing institutional and international arrangements as well as the deployment and availability of infrastructure and the political and socio-economic environment (7). People's attitudes will play a major role in this and the paper provides insights from Sydney's Gen Z.

## Materials and Methods

This exploratory survey of adult Gen Z is based on an online questionnaire containing some quantitative and some qualitative questions. We chose to conduct the survey online because of the target group's familiarity with using the internet. The survey offers an opportunity to explore the views of young people using inexpensive, completely voluntary, interactive, without data restrictions and open in nature method. Tailored to the main characteristics of the target population, the research aimed to develop a first-hand understanding about a specific group faced with a particular situation which we believed was worth exploring but about which there was no prior knowledge (32). With Gen Z being the first all-digital generation, we took an open-mind approach without any explicit expectations to try to understand what excites and affects them (33) in relation to cultured meat.

The quantitative part of the questionnaire requested demographic data about the participants while the qualitative components focused on collecting their opinions related to cultured meat. There were five sections in the questionnaire:

1. Demographic data related to age, gender, employment status, profession, and income level;
2. Dietary preferences based on frequency of meat consumption, ranging from daily to a few times per week, occasionally and never;
3. Opinion about cultured meat and whether it is normal and necessary to accept and if available, consume cultured meat;
4. Preference for different meat alternatives, namely insects, plant-based options and cultured meat;
5. Factors and reasons which may influence people to embrace new meat alternatives.

All participants were recruited randomly, electronically from a pool of 30,000+ names registered in a database established by the researchers. They used a checkbox on the questionnaire to indicate their informed consent to take part voluntarily in the survey. An ethics approval was obtained from Curtin University Human Research Ethics Committee. The response rate to the survey was 75% which is relatively high. Such a response rate is considered good to eliminate potential bias from young people who have responded and those who have chosen not to respond (34).

The data gathered from the qualitative sections were in the form of free verbatim comments and direct quotations. As with most qualitative studies, we continued collecting additional data until a saturation of results was achieved, meaning the data no longer provided any further clarity or insights related to the explored topic (35). The collected data were analyzed both manually using researcher discretion and with the help of the computer-assisted qualitative data analysis software NVivo11 (36). Frequently occurring expressions and themes were coded to produce manageable categories related to the topic of cultured meat.

## Results

Conducted in 2019, this exploratory study of adult Gen Z in Sydney, Australia covered 227 (*n* = 227) participants born between 1995 and 2001. Below we present the demographic and dietary description of the sample followed by the respondents' opinions about cultured meat.

### Description of the Sample

In total, 227 representatives of Sydney adult Gen Z participated in the study through voluntary self-selection (see Table 1). The share of male participants (55% or 125 men) was higher than that of female respondents (45% or 102 women). This gender difference in favor of male participants is relatively small and although it was not deliberate, it was important to capture sufficiently men's views as previous research shows that they more frequently opt for meat options (5). There were no major differences in the opinions presented by the male and female participant groups and therefore, the analysis to follow does not apply a gender lens.

**Table 1:** Demographic characteristics of Sydney Gen Z sample.

**Demographic parameters**	**Category**	**Total number (*****n*** **=** **227)**	
			%
Gender	Male	125	55%
	Female	102	45%
Age	18 years	18	8%
	19 years	19	8%
	20 years	39	17%
	21 years	33	15%
	22 years	40	18%
	23 years	42	22%
	24 years	36	13%
Household income	Under $50,000	62	27%
	$51,000 to $74,000	77	34%
	$75,000 to $100,000	52	23%
	$101,000 or more	36	16%
Employment	Full time	114	50%
	Part Time	96	42%
	Study	17	7%

The sample is statistically representative at a 95% confidence level with an acceptable margin of error within 5.2%. Table 1 shows the break-down of the participants by age. The average age of the sample is 21.4 while the median age is slightly higher at 22.

Overall, the sample consists of relatively young adults, the majority of whom have transitioned to being economically independent with 50% being in full-time employment, 42% working part-time and only 7% studying (see Table 1). The average household income of the Sydney adult Gen Z sample is estimated at around $71,000 per annum.

**Figure 1:**
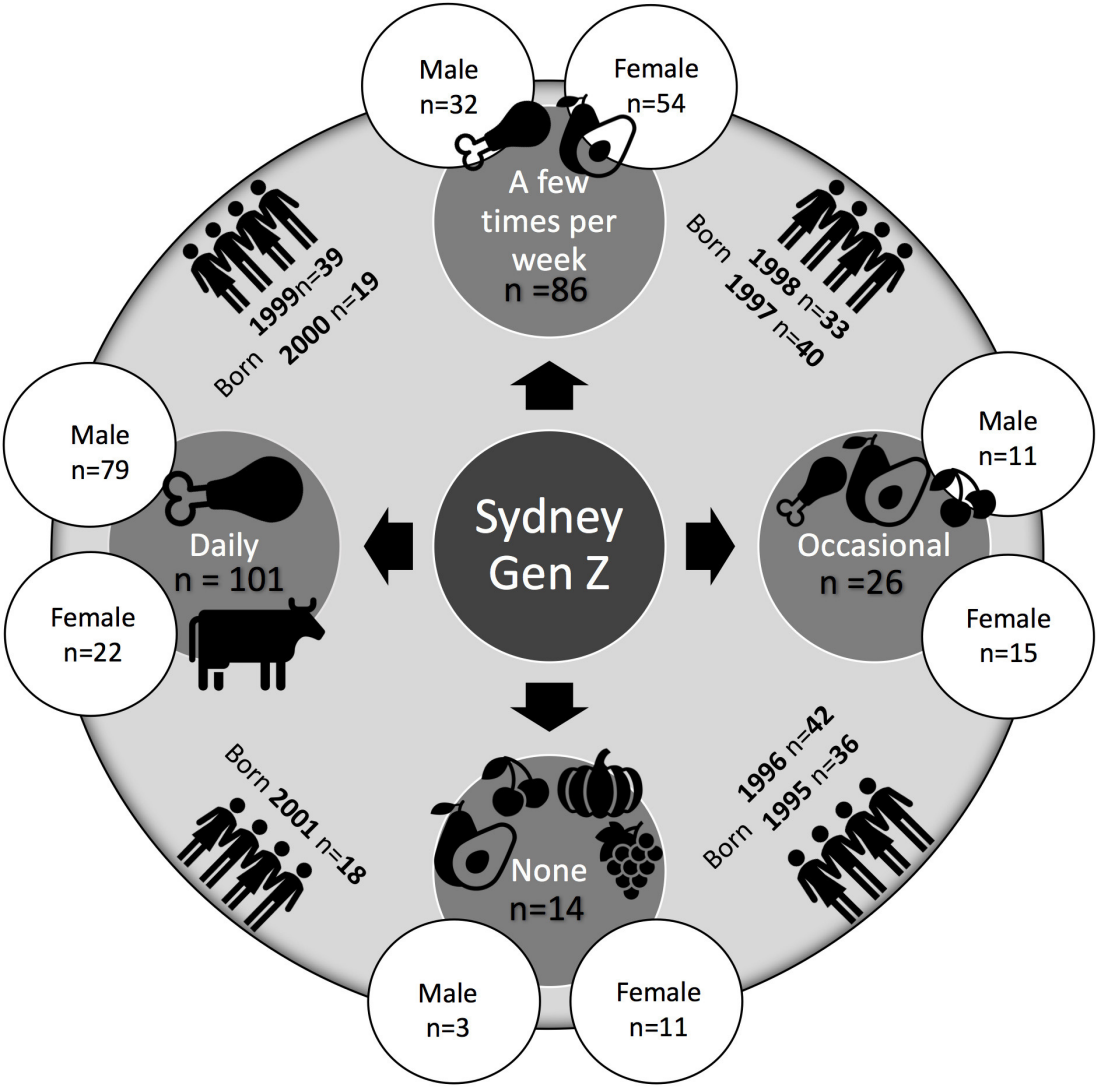
Meat consumption of Sydney Gen Z sample.

The levels of meat consumption varied significantly amongst the study's participants (see Figure 1). However, there was explicit preference for meat in line with the general trends in Australia which has one of the highest per capita meat consumptions in the world (37). The majority of the respondents, namely 44%, consume meat on a daily basis, followed by those who eat meat a few times per week−38%, occasionally−10%, and never−6%. A 2019 study of the Australian population shows that the food of 12.1% of Australians (or 2.5 million) is all, or almost all, vegetarian (38). Our sample shows a lower percentage of strict vegetarians (6%) but the share of those who eat meat occasionally or have excluded meat completely from their diets, is higher at 16%. Previous research shows that vegetarianism is also constructed as a social category with some vegetarians occasionally consuming meat (39). Hence, a possible explanation for the lower rate of full vegetarianism in our sample is the fact that we asked about frequency of meat consumption rather than how people self-identify. These considerations allow us to conclude that the survey sample is not that different from the overall food trends in Australia.

**Figure 2:**
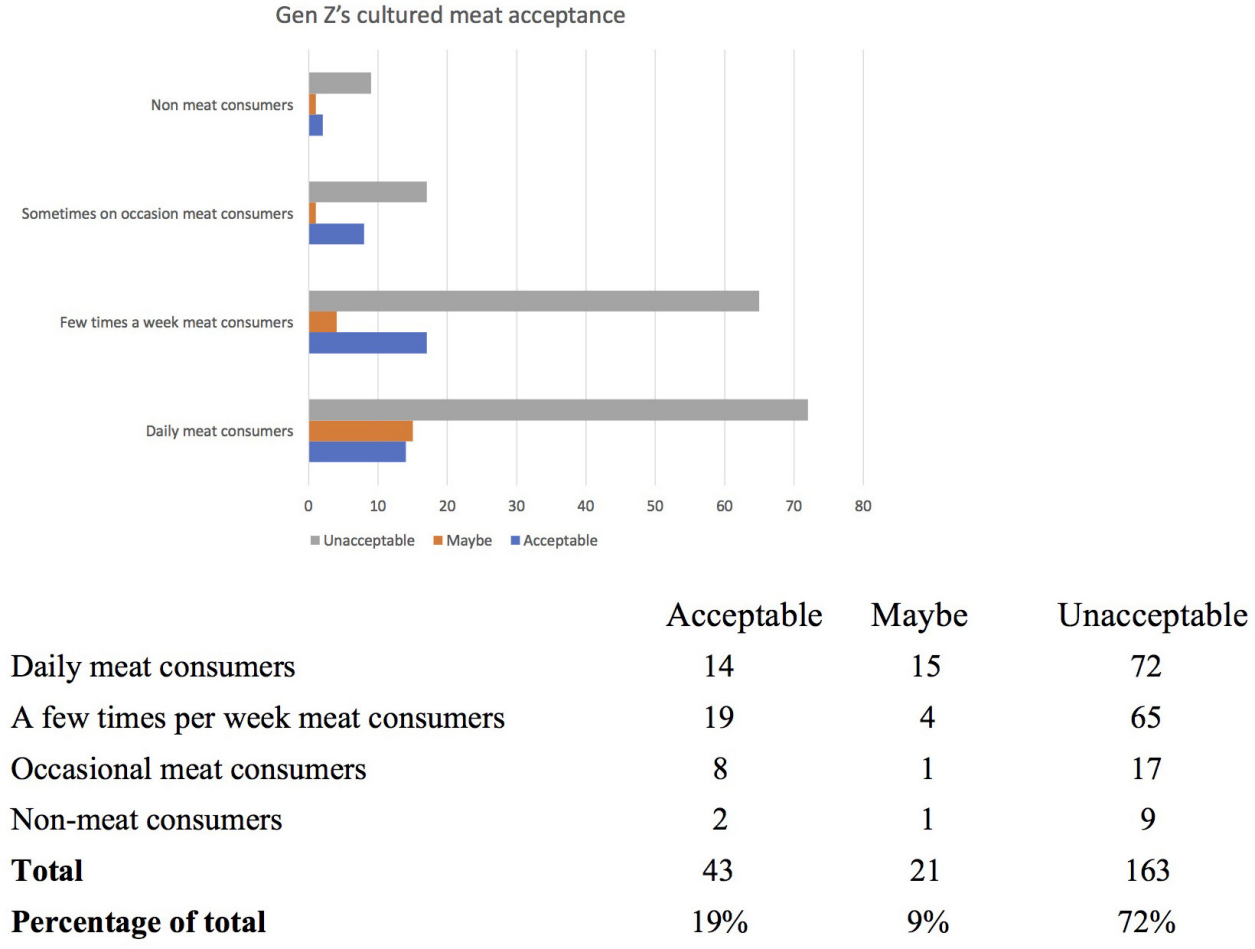
Acceptance of cultured meat by Sydney Gen Z.

Only 97 participants (or 43% of the sample) believed that there is a need to replace traditional meat with other food alternatives, including plant-based options and cultured meat. This shows a relatively low level of awareness amongst Australian youth about the negative environmental and other impacts of livestock. Furthermore, cultured meat is not seen as an attractive alternative with only 19% (or 43 participants) accepting it as a food option and a further 9% (or 21 people) being hesitant. The remaining 72% (or 163 participants) were categorically of the opinion that cultured meat is not acceptable to them (see Figure 2). What are reasons for these low levels of acceptance of cultured meat are discussed in the qualitative analysis below.

### Attitudes Toward Cultured Meat

Concerns dominated Gen Z's attitudes toward cultured meat. They included personal concerns, related to anticipated taste, health and safety, as well as societal considerations related to whether we need to accept the need to consume cultured meat and whether it is more sustainable. Socio-cultural impacts, such as perceptions about masculinity and animal meat being an Australian pride, were also raised. Some were concerned about the animals themselves. Finally, there were those who saw cultured meat as a conspiracy orchestrated by the rich and powerful and were determined not to be convinced to consume it.

This wide range of opinions is discussed below using excerpts verbatim from the survey respondents. It appears that there were overwhelming negative attitudes although some were prepared to give cultured meat a try.

#### Personal Concerns

Gen Z expressed many personal concerns related to cultured meat. They included disgust about the anticipated taste and eating experience as well as health and safety concerns.

##### Disgust

When it comes to food, and novel food in particular, people's first reaction is related to the anticipated taste. The study's participants were divided in their reaction. A large majority of the Sydney Gen Z respondents (*n* = 163, 72%) associate cultured meat with a feeling of uneasiness and discomfort. This is expressed with statements, such as: “It makes me really sick,” “it's really disgusting,” and “I may vomit. Sorry” (see Table 2). Some (*n* = 64, 28%), however are more intrigued: “I am open to try it,” “if it's dressed up nicely and appetizing” and “the more we know about the process behind cell-based meat production the better acceptance we may have” (see Table 2).

**Table 2:** Sydney Gen Z's disgust and willingness to try cultured meat.

	**Disgust**	**Willingness to try**
1	“*I am more pro meat than pro other meat alternatives including in vitro. When I think about is, I feel disgusted. It makes me really sick*.” (daily meat eater, barista, age group 18–20 years)	“*I don't know much about in vitro meat. Humans pretty much eat anything. If it's dressed up nicely and appetizing, I'm all for it. I love to try cultured meat. Life's too short to eat bland and uninspiring, lol*”. (daily meat eater, project manager, age group 21–24)
2	“*What is so clean about this meat, nothing clean. It's full of chemicals and looks disgusting. It's really disgusting. I won't eat it*.” (daily meat eater, hospital attender, age group 21–24 years)	“*Many of my peers will say it's not normal to eat cultured meat, but I am open to give it a try. It looks good. You can say something is not good if you never try it*.” (daily meat eater, assistant accountant, age group 21–24 years)
3	“*Definitely not normal for me and my family. I feel sick even to think about eating meat produced from stem cells. Totally sick*.” (daily meat eater, pizza maker, age group 18–20 years)	“*Meat has been linked many times to several diseases. I believe trying in vitro option that is clean from these diseases could be good for the humanity*.” (daily meat eater, boarding flight assistant, age group 21–24)
4	“*Cultured meat, insects are not normal, even disgusting to some extent. We won't eat it and the idea of it is sending messages of pure disgust to me*.” (a few times per week meat eater, technical assistant, age group 21–24 years)	*Cultured meat has been there for a while, but still in development stage only, not on the market. We never try it and we don't know what its taste [is] like. I am interested to try it and I reckon the more we know about the process behind cell-based meat production the better acceptance we may have*.” (a few times per week meat eater, university student, age group 18–20 years)
5	“*Cultured meat seems weird and a bit disgusting, not because of its look, but because the way it's made*.” (a few times per week meat eater, personal assistant, age group 21–24 years)	“*We will need lots of adapting before putting cultured meat into our mouth. Maybe if it looks like the real thing and if we don't know what it is we will be all willing to eat it*.” (a few times per week meat eater, investment coordinator, age group 21–24 years)
6	“…*I don't think we will be able to overcome our aversion to laboratory-grown meat. It's disgusting to grow something from a piece of tiny cell*.” (a few times per week meat eater, finance officer, age group 21–24 years)	“*I have no knowledge about cell-cultured meat to comment on it. But if it is helping with animal welfare, I believe people that consume meat should be open to try it. They can reduce the animal burden to die to feed them*.” (non-meat eater, bank cashier, age group 21–24 years)
7	“*In vitro meat is much less natural than normal meat. It looks really disgusting and the thoughts we should eat it in the future are making it even more revolting and provoking high unacceptance and dislike*.” (a few times per week meat eater, DJ and Uber driver, age group 18–20 years)	“*Lab grown meats. I am open to trying as they sound interesting*.” (daily meat eater, drafter, age group 21–24 years)
8	“*I always try to be open minded, but these alternatives are not so appealing, I have no problems with veggie-based meat, but with the larvae and crickets, and cultured meat I may vomit. Sorry*.” (a few times per week meat eater, club team leader, age group 21–24 years)	“*I will try cultured meat if available and of curiosity, but I usually will go with real meat*.” (daily meat eater, supply chain coordinator, age group 21–24 years)

##### Health and safety

Gen Z is very much nutritionally aware and these young people are committed to healthy eating and wellness (40) while open to exploring new food choices which often were not available to previous generations in Australia. It is not surprising then that the Sydney Gen Z is casting doubts whether cultured meat is a healthy and nutritional dietary option with a third of them (*n* = 73, 32%) believing this not to be the case. They described cultured meat as being “far too chemically processed,” associated with “engineering and modifications.” Others, however, are of the opinion that “it should be healthy and nutritious if they get it right.” There are also those who frankly admit: “I have absolutely no idea… whether these alternatives… are nutritious like real meat” (see Table 3).

**Table 3:** Sydney Gen Z's perceptions about healthiness and safety of cultured meat.

	**Healthiness**	**Safety**
1	“*In vitro meat is overly processed. In our society, at school, uni, media, magazines, articles, everywhere we are told to limit the consumption of processed food*.” (daily meat eater, sports coach, age group 18–20 years)	“*Cultured meat is an interesting option. Experimentation can lead to great findings for broadening humanity's ever developing knowledge. Although, I think it could be a game not with good end and lots of adverse effects for the humanity, because of our greed for meat*.” (non-meat eater, yoga teacher, age group 21–24 years)
2	“*There is a trend now people to become flexitarian and to eat meat alternatives, but all these including cultured meat are not healthy. I prefer to reduce meat intake but will not eat these modern things*.” (daily meat eater, university student, age group 18–20 years)	“*If we think about the future food security of the planet, we have to be ready to accept anything. But I believe engineered and chemically processed food are not good for human to consume. I even think these will counteract in the opposite direction and contribute to human non-communicable diseases*.” (occasional meat eater, nurse, age group 21–24 years)
3	“*You can't have ribs, steaks etc. out of fake meat and it's not appealing. Even in the future the scientists can grow these, it will be far too chemically processed to be normal and healthy thing to consume*.” (daily meat eater, mathematics tutor, age group 18–20 years)	“*Maybe there are more health benefits to not eat meat than eating cultured meat. They could be some future side effects to human from eating it. It's good that it is not mass market produced yet*.” (non-meat eater, solicitor, age group 21–24 years)
4	“*No idea how normal meat will be sourced from a lab instead of a farm. More likely not good and unhealthy for us to consume. I will incline toward opting it out*.” (daily meat eater, university student, age group 18–20 years)	“*Artificial growth cells and hormones to make it edible in vitro meat thanks god that is still an underdeveloped technology. No one knows what this meat will be lacking and what will be the side effects for us*.” (daily meat eater, high school sports aid, age group 18–20 years)
5	“*Not sure why we should think of meat substitutes as healthy. They never will be healthy and good for you like plain fruit and veggies. See the cultured meat, plant-based engineered burger. People will always associate them with engineering and modifications*.” (a few times per week meat eater, hairdresser, age group 21–24 years)	“*Not normal, maybe the good thing about it is that humans created some emerging modern technology but multiplying cells to grow meat for human is wrong. It's against the nature and if we consume it, we will pay sooner or later for this*.” (daily meat eater, university student, age group 18–20 years)
6	“*Necessary with respect to the environment and the animals, but it's unknown how healthy cultured meat is for humans to consume on a regular basis like meat. More likely not that healthy having in mind the way it's produced*.” (a few times per week meat eater, office assistant, age group 21–24 years)	“*Scientists created in vitro meat cultivation because of their interests to advance in technologies, but this doesn't mean what they created is good for human consumption without any future negative effects*.” (daily meat eater, trading operations analyst, age group 21–24 years)
7	“*A replacement for meat with in vitro – the scientists are trying hard to replicate real meat, so it should be healthy and nutritious if they get it right*.” (a few times per week meat eater, administrator, age group 18–20 years)	“*Need scientifically proven information about cells-made meat before trying it. It could have some unhealthy side effects*.” (occasional meat eater, graphic designer, age group 21–24 years)
8	“*In vitro mimic the taste, texture and protein content of meat. Honestly, I have no idea how good it is for you. I have absolutely no idea whether these alternatives are having similar iron, zinc and magnesium content to say if they are nutritious like real meat. I'll say they are fake and not healthy for us to eat.”* (a few times per week meat eater, office administrator, age group 21–24 years)	“*We don't know yet if we are going to eat cultured meat. It's still in early stage of its development and far away from the natural meat appearance. It can't be possible to not have some future negative effects on human*.” (daily meat eater, physiotherapist, age group 21–24 years)

Although sometimes it is difficult to strictly draw the boundary between food quality and food safety, the latter refers to the way the products we consume are being handled and whether there are any negative health consequences from consuming them. Technically, cultured meat is expected to be produced within a clean, sterile and highly controlled environment to prevent any food-related risks. Nevertheless, Gen Z are not convinced that it will be safe for consumers. A major worry for them are the possible unknown “adverse,” “negative,” “hidden side effects” of cultured meat (see Table 3). This resembles some of the concerns expressed in relation to the consumption of insects in our previous study (29).

#### Societal Concerns

There are two broader societal themes that emerged in the respondents' answers. One is related to food availability and the other to the environmental impacts of the current meat production. This comes against a background where only a third (*n* = 74, 33%) of the Gen Z participants are willing to change their meat-related behavior, with that share amongst daily meat consumers being much lower (*n* = 18, 18% of the group of daily meat eaters).

##### Food availability

When it comes to the question about food availability, opinions within the survey sample were divided. Many (*n* = 57, 25%) saw the need to accept cultured meat because of population growth or inability to produce enough livestock-based meat. Expressions, such as “not enough meat for everyone” and avoid “a food war” (see Table 4) were used. On the other hand, there were many voices which similarly recognized the need to look at human diet but did not see cultured meat as an option to feed the world or reduce the food's impact on the environment. Examples include: “*in-vitro* meat is unwanted,” “it's like a Frankenstein creation” and “can we just eat fruits and veggies?” (see Table 4).

**Table 4:** Sydney Gen Z's perceptions about need for cultured meat.

**N**	**Necessary**	**Unnecessary**
1	“*With the population increase it will be very, very necessary to eat meat substitutes and clean meat. I hope there is not going to be a food war*.” (daily meat eater, assistant manager, age group 21–24 years)	“*For me is totally unnecessary to push ourselves to eat artificial, cultured meat. Don't we have access to plenty of meat at the supermarket?! Why should we invent the already invented?*” (daily meat eater, community services, age group 21–24 years)
2	“*Solution for meat replacement like lab meat, insects, plant-based are weighty. Availability is important for something to become necessity*.” (daily meat eater, swimming coach, age group 18–20 years)	“*I feel like it makes sense for us to move away from consuming meat now that we hear of its negative effects on the environment, but with growing lab meat we will still continue to eat meat. What's the point of lab-growing it? It's not needed*.” (daily meat eater, human resources officer, age group 21–24 years)
3	“*I'll see it necessary to grow meat from cells when there are not enough cows to graze in Australia. Right now, we are enjoying meat and what scientists are up to is something that we will deal with when we reach the point to need it*.” (daily meat eater, early childhood teacher, age group 21–24 years)	“*Products that are simulating meat-based dishes but are made from alternate, possibly more sustainable, sources are fine, but I am not sure yet about in vitro meat. It's unnecessary, and sounds very scary*.” (daily meat eater, teacher, age group 21–24 years)
4	“*Cultured meat is produced heavily with chemical engineering, but there is no doubt that things like it will be very necessary for the future when there maybe will be not enough meat for everyone*.” (daily meat eater, customer service officer, age group 21–24 years)	“*It will be a necessity in the future to consume some substitutes as we will face lack of resources, but in vitro meat is unwanted. It is artificially produced*.” (a few times per week meat eater, online coordinator, age group 21–24 years)
5	“*I think it is necessary to produce different type of meat – cultured, plant-based etc. If we want to reduce our footprint on the planet and the harm to animals, we should accept it*.” (daily meat eater, disability support worker, age group 21–24 years)	“*The space and resources needed to farm meat will be under significant pressure in the future, and ethical obligations will prevent factory style farming. But cultured meat farming is a wrong thing to do. It's like a Frankenstein creation, with the difference that we are going to eat it. Sounds not right.”* (a few times per week meat eater, 24 traffic control, age group 21–24 years)
6	“*Increasing demand for food and decreasing space will push out appetite for alternative food, so anything on offer cultured meat, plant-based things will be fine as long as they're affordable and available. Alternatives are required to feed people in the future*.” (a few times per week meat eater, personal assistant, age group 21–24 years)	“*We are worried and focussed in the future. It is absolutely necessary the humanity to think of new alternatives to meat, but I am not sure why we should focus on creating more processed meat if this is what we have to reduce from our plates. Can we just eat fruits and veggies?*” (occasional meat eater, IT programmer, age group 21–24 years)
7	“*Necessary because of climate change and because of animal suffering. It is good that scientists are making clean meat without using the animals*.” (a few times per week meat eater, sales assistant, age group 18–20 years)	“*The meat industry is one of the greatest contributors to global warming - something that needs to be addressed. In vitro meat grown from cells is not a cool option. It's another way to produce more meat.”* (non-meat eater, massage therapist, age group 21–24 years)
8	“*I personally believe that using more meat alternatives, mostly plant-based even insects and in vitro meat will have a positive outcome for the environment, humanity and animals. These are all needed for securing future food*.” (a few times per week meat eater, retail operator, age group 18–20 years)	“*Even we do have enough information, if we want to save the planet and reverse the climate change, we don't need to grow more meat, especially artificial like in vitro as we can eat veggies and be happy*.” (occasional meat eater, dog groomer, age group 21–24 years)

##### Environmental impacts

The need for switching to meat alternatives and more sustainable food choices was highlighted by a large number of study participants (*n* = 93, 41%). They were however unsure whether cultured meat is better and was described as “resource consuming” and not being “environmentally friendly” (see Table 5). It is also interesting to note that these concerns were not raised by those who consume meat on a daily basis.

**Table 5:** Sydney Gen Z's environmental concerns related to meat.

1	“*Livestock producers must make sure that livestock is environmentally sustainable. Ideas like growing meat on a plate under shelter is quite unsustainable*.” (a few times per week meat eater, business owner, age group 21–24 years)
2	“*With the projected rapid decline in meat availability because of climate change, it's important to be substituted with some meat alternatives but not cultured meat. You can't ensure livestock and environmental sustainability with producing extra meat which is the cause of the problem.”* (a few times per week meat eater, bartender, age group 21–24 years)
3	“*In vitro meat and other alternatives are important as it can help to reduce greenhouse emissions, save animals and focus on health*.” (a few times per week meat eater, installer, age group 21–24 years)
4	“*Lab meat could minimize the associations with the environmental impacts and ethical issues, but it is still resource consuming. Think about how much energy is put into it being under constant light and in a special environment. It's not a sustainable option*.” (a few times per week meat eater, remedial massage therapist, age group 18–20 years)
5	“*We need to look after the environment. Lab-meat is environmentally better than livestock produced meat and better for the animals*.” (occasional meat eater, acrobatics coach, age group 21–24 years)
6	“*I believe that at the rate our planet is going, we will all have to consider eating less meat. Eating more alternatives for a sustainable diet, like even adopting Meatless Monday, including less meat in our diets or eating more plant-based options. I can't see easily cultured meat fitting into this*.” (occasional meat eater, receptionist, age group 21–24 years)
7	“*I'm concerned for the environment and our resources. But I rather eat fruit and veggies than cultured meat or other men-made meat substitutes*.” (occasional meat eater, youth worker, age group 21–24 years)
8	“*I don't consume meat but in principle meat alternatives should be sustainable and environmentally friendly (which meat-free alternatives does not necessarily equate to). I don't think cultured meat is any of those*.” (non-meat eater, finance officer, age group 21–24 years)

#### Socio-Cultural Concerns

Two main socio-cultural considerations emerged from the survey. The first is related to the perceptions about masculinity and that meat is the men's choice, while the second is about Australia priding itself as a producer of quality animal-based foods, such as beef.

##### Masculinity

The majority of the male daily and a few times per week meat consumers (*n* = 58, 52% and *n* = 58, 26% of the sample) found cultured meat threatening to their manly traits. Similar concerns have been expressed in previous studies (28, 29), indicating that many men are not ready and unwilling to contemplate changing their dietary preferences (5, 41). Table 6 shows the expressions such men use, e.g., “rip meat from the bone,” “real men eat meat,” and “I don't think is appropriate for me to eat cultured meat if there is a real, bloody tasty meat around.”

**Table 6:** Sydney Gen Z's perceptions of masculinity related to meat.

1	“*I can't abandon my meat for other food, even grown from the real animal tissue. A man like me prefers to rip meat from the bone with teeth, to feel the taste, the smell, the blood before I cook it on a barbie, the goodness of the real juicy meat*.” (daily meat eater, project officer, age group 21–24 years)
2	“*Man, like me needs good nutrients. Fake meat does not have the same amount of nutrients, minerals, vitamins the actual meat has. Would the protein be the same? I doubt it. I will stick with the manly meat diet*.” (daily meat eater, brick layer, age group 18–20 years)
3	“*There is a strict men's rule saying real men eat meat, not artificial meat and I will stick to this rule*” (daily meat eater, IT programmer, age group 21–24 years)
4	“*There are no bones sticking out of lab meat. It's blended and super fake. No one maintaining his masculinity will want to eat a fake meat without a bone*.” (daily meat eater, university student, age group 18–20 years)
5	“*In vitro meat is not a food for genuine men. I think you should eat manly things as part of a well-balanced masculine diet and in vitro is absolutely not one of them*.” (a few times per week meat eater, clinical associate, age group 21–24 years)
6	“…*Also as a man, I don't think is appropriate for me to eat cultured meat if there is a real, bloody tasty meat around*.” (a few times per week meat eater, song writer/installer, age group 21–24 years)
7	“*Forget about edible insects, lab meats, processed plant burgers and other nonsense they try to introduce us to eat. I feel only the juicy meat is natural for my body and health, so I eat it regularly and I feel pretty bloody manly*.” (a few times per week meat eater, personal assistant, age group 21–24 years)
8	“*Not interested in these in vitro or other processed plan-based foods. They aren't natural, nutritious, they are not masculine food for Australian men or other men in the world*.” (daily meat eater, registrar, age group 21–24 years)

##### Australian pride

Pride in Australia being a producer of “superb quality” and “best in the world” meat (see Table 7) where it is widely available and affordable was expressed by some participants (*n* = 29, 13%). Many stress that “meat is plentiful in Australia” and “we produce lots of good meat the nation is proud of” (see Table 7). These participants see cultured meat as a disloyalty to Australian meat and betrayal of their country.

**Table 7:** Sydney Gen Z's pride with Australian meat.

1	“*Coming from a meat-eating nation with one of the best superior quality meats in the world, I feel we should be quite cautious not to betray our beautiful meat for this artificial meat*.” (daily meat eaters, shop assistant, age group 18–20 years)
2	“*I believe in vitro meat and other plant-based meat are not that essential, meat is plentiful in Australia and one among the best in the world. We don't need to worry too much*.” (a few times per week meat eater, legal secretary, age group 21–24 years)
3	“*In Australia we produce lots of good meat the nation is proud of and more lab-grown meat is unnecessary*.” (non-meat eater, yoga instructor, age group 21–24 years)
4	“*Aussie meat is the best and part of our culture, no any other meats, even lab meat, can replace its quality*.” (daily meat eater, electrician apprentice, age group 18–20 years)
5	“*We love Aussie meat. The best in the world and I can't replace it with gross lab meat*.” (daily meat eater, planner, age group 21–24 years)
6	“*Australia grows naturally exceptional livestock and produces the best meat cuts worldwide. Not clear how the lab meat is grown, what chemicals, preservatives they put into it to prevent it from rotting or to maintain its taste, texture*.” (daily meat eater, clients' relations, age group 21–24 years)
7	“*Not normal for me to eat some fake, synthetic meat, especially living in Australia where the meat is with no doubt the best in the world*.” (daily meat eater, pastry chef, age group 21–24 years)
8	“*Right now, it is not natural at all to consume lab meat. It looks yuck, patty not like meat. It seems quite artificial and can't even compete and beat the Australian meat which is number one*.” (daily meat eater, laborer, age group 21–24 years)

#### Concerns About Livestock Animals

Concerns about animal welfare and dignity were expressed by some of the participants in the Sydney study. They saw cultured meat as a way to avoid animal suffering but others also raised concerns that using animal cells for growing meat in a lab is unethical from the point of view of the animal.

##### Animal welfare

Improved animal welfare because of a switch to cultured meat was seen as an advantage by some participants (*n* = 37, 16%). They described this as “this way we are not harming the animals,” “stop exploiting them” (see Table 8). Such voices were raised mainly by those who consume meat less frequently.

**Table 8:** Animal welfare concerns by Sydney Gen Z.

1	“*Extremely necessary as we have not enough resource to sustain the planet and this way we are not harming the animals, but they are still helping us to eat using their cells to grow meat*” (a few times per week meat eater, recruitment agent, age group 21–24 years)
2	“*Very necessary for climate change and for the animal suffering. I believe scientists [have] done it with all these considerations in mind.”* (a few times per week meat eater, assistant manager, age group 21–24 years)
3	“*With in vitro you don't need to kill animals to source your meat. This makes people feel good about the animals*.” (a few times per week meat eater, administrator, age group 21–24 years)
4	“*In vitro is good for animal welfare viewpoint. Other than that, it's still an imitation*.” (a few times per week meat eater, kindergarten aid, age group 18–20 years)
5	“*It's good for the animals not to be exploited for human food, but actually if the humans reduce their consumption of meat there is no need of a huge exploitation. We don't even need inventions like cultured meat, just change of our diet will sort the issue*.” (a few times per week meat eater, café staff, age group 18–20 years)
6	“*It's needed source of meat without harming animals. I believe it's humane way to produce lab grown meat instead of real meat*.” (occasional meat eater, carpenter, age group 18–20 years)
7	“*I don't like in vitro and plant-based meats as I care about the animal welfare and don't want to consume anything that resembles meat*.” (occasional meat eater, project officer, age group 21–24 years)
8	“*Cultured meat is not applicable to my diet. We have to be ethical to animals and stop exploiting them, not artificially multiplying them*.” (occasional meat eater, community support worker, age group 18–20 years)


##### Animal dignity

Another interesting nuance of the ethical use of animals for food production was the issue about animal dignity raised by some participants (*n* = 26, 11%). These participants represented different dietary practices and they spoke about “the permission of the cow” to use its cells, being “really unethical and painful” (see Table 9).

**Table 9:** Animal dignity concerns by Sydney Gen Z.

1	“*Artificial meat substitutes are unnatural. You could figure it out with a simple Google search. And clean meat is not even that clean as it is against the animal dignity to be grown from a cell*.” (daily meat eater, engagement coordinator, age group 21–24 years)
2	“*If these are environmentally and ethically produced, they will be good to eat and when people have more knowledge, they could consume them on a regular basis. The problem is that they are not ethically produced with the permission of the cow…*” (daily meat eater, university student, age group 18–20 years)
3	“*I am skeptical about lab-meat. I think we need to work hard to mimic the real meat, but in an ethical for the animal way*.” (daily meat eater, soccer coach, age group 18–20 years)
4	“*I hope it is not becoming necessary to eat only cultured meats. I read that the cells are drawn from live animals and then manipulated chemically to grow. It's quite strange thing to do. It is sad thing to do and really unethical and painful*.” (a few times per week meat eater, waitress, age group 18–20 years)
5	“*Humans need to look at new protein sources but not to cultured meat. It is unnatural meat made without even asking animal for a consent*.” (non-meat eater, assistant IT software engineer, age group 21–24 years)
6	“*Not exactly made from animals, but from animal cells. This sounds really scary and as vegetarian and animal activist I think this is also another way to be unethical to animals, creating their own counterparts like AI, robots*.” (non-meat eater, office worker, age group 18–20 years)
7	“*Humanity must meet the future generation's needs. In vitro is giving new perspectives to grow meat without the animal and with respect for the animal*.” (non-meat eater, dancer, age group 18–20 years)
8	“*I have never eaten cultured meat. I think it is still very expensive to produce and not yet fully developed as a technology. Plus, the technology itself is creating synthetic, artificial products using animal cells with no respect for the animal dignity*.” (a few times per week meat eater, dental nurse, age group 21–24 years)

#### Conspiracy Concerns

Concerns were raised by Gen Z whether cultured meat is part of some hidden agenda, including those who have funded its development wanting to see return on their investment. The participants were divided in two groups—pro and against the sponsors of cultured meat (see Table 10). Those who are against the development of cultured meat describe this as “another thing our generation to worry” and explain that “there must be serious interest from people who created it.” By comparison, those who support this innovation describe it as “money… invested for a good cause,” “a smart move” by people who are “pretty advanced thinkers.”

**Table 10:** Sydney Gen Z's attitudes toward investors in cultured meat.

	**Negative**	**Positive**
1	“*When I first heard media reporting something about billionaires like Gates and Branson supporting cultured meat R and D, I remember I said to myself this is another thing our generation to worry. We are not interested how much they put into it. We've seen enough of this 'business deals' and fake 'benefits' for us, the normal humans. I worry a lot*.” (daily meat eater, university student, age group 18–20 years)	“*I'm not sure. I don't have enough information and I am not a specialist. But I read that scientists and rich people like Bill Gates and Richard Branson are investing in cultured meat and it must be good as these people are not investing their money for something that is not going to work and them to profit from it*.” (daily meat eater, cashier, age group 21–24 years)
2	“*Rich people like Branson and Gates have invested in this type of fake alternatives and now they are looking to have a return on investment and are marketing cell-based meat as good replacement of meat. Australians have enough good cuts of meat to consider eating cultured meat. No FOMO [fear of missing out]. None of my friends will do*”. (daily meat eater, army force soldier, age group 21–24 years)	“*We have to be open to new food sources because we have no idea what food we will have available in the future. If we learn how to produce meat from animal cells, can you imagine how much meat we can produce from a single cow? We can even produce only the best cuts*.” (a few times per week meat eater, cinema manager, age group 21–24 years)
3	“*I am not trying cultured meat even for free. Who came up with the idea for this thing? Who stays behind this? There must be serious interest from people who created it, in a similar way like pharmaceutical companies*.” (daily meat eater, veterinary clinic assistant, age group 21–24 years)	“*I think it's positive that high profile people invest in cultured meat. I think their moneys are invested for a good cause*.” (a few times per week meat eater, carpenter, age group 18–20 years)
4	“*Lab meat is a huge bioengineering animal cells made thing and not for a good cause. There are people with big money behind lab meat and obviously want to profit from it. I am sick of people not seeing the proper root cause of our dietary problems. We need not another meat but reduction of what we eat right now*.” (a few times per week meat eater, client liaison officer, age group 21–24 years)	“*Meat from cow tissues and cells is a fascinating concept closer to the sci-fantasy movies than to our reality. I know many wealthy people invested in the technology to develop this kind of meat, but the normal people are not familiar with it yet. I think it's a good thing to do as humanity needs to use the advancement of the technology*.” (a few times per week meat eater, project officer, age group 21–24 years)
5	“*It's a new technology still and it doesn't mean that in vitro meat is good to consume if it doesn't involve real livestock. The people that financed it claimed it as a huge discovery for the future humanity food security. Same way soon we will start growing humans from cells. What a hypocrisy*.” (daily meat eater, landscaper, age group 21–24 years)	“*People think they are natural and good because we don't kill animals. I need more information about lab grown meat is kind of needed. I feel the people that gave the money for this lab meat are pretty advanced thinkers*.” (a few times per week meat eater, office manager, age group 21–24 years)
6	“*I heard and believe clean meat is part of big corporations' vast interests. It has nothing to do with meat scarcity or environment issues. It's part of another big scheme, like the production of drugs*.” (a few times per week meat eater, business development assistant, age group 21–24 years)	“*I think making alternatives like lab meat, plant-based that are not harming the environment and the animals is a smart move. I think lab meat was sponsored from Bill Gates*.” (occasional meat eater, practice manager, age group 21–24 years)
7	“*Cultured meat is another food part of some billionaire's interests. I think this was backed by Sergey Brin not long time ago. People with money think they can buy everything, but they can't buy us or make us eat their fake meat investments*.” (a few times per week, business project coordinator, age group 21–24 years)	“*We are so cruel to animals and I think this is why rich philanthropic people, scientists are creating the lab meat to save them from suffering*.” (occasional meat eater, human resources officer, age group 21–24 years)
8	“*Despite the ambition of Microsoft Gates and Google Sergey Brin I don't think people will consider cultured meat as a normal meat from real animals. I am wondering whether something unpleasant behind its production is holding it from mass consumer release*.” (a few times per week meat eater, technician, age group 21–24 years)	“*As people are becoming more socially aware of the consequences of eating meat, in vitro meat and other alternatives will become popular. This is what I think will happen, especially from my generation point of view. It's good that they have stable finance providers and supporters*.” (non-meat eater, university student, age group 18–20 years)

### The Future of Cultured Meat

The respondents were also asked their opinion about the future place of cultured meat in human diets through three different perspectives: first, how they see its future; second, in relation to other meat alternatives; and third, what would make them accept alternatives to traditional animal meat. Their answers are presented in turn below.

#### Prospects for Cultured Meat

Lack of information about the way cultured meat is created, the substrates and the processes used combined with it not being yet available in Australia, was making it an undesirable food option for many of the respondents (*n* = 103, 45%). This was an interesting observation given the fact that Gen Z is accustomed to be using the web for communication and thrives in the social media. A further large group (*n* = 88, 39%) was indecisive about the future of cultured meat. The remaining participants (*n* = 36, 16%) saw a good value in cultured meat, believing that if it is done right it could work and become one of the “future food trends.” Table 11 presents some examples of the way the participants described cultured meat becoming a normal food choice or continuing to be perceived as abnormal, unnatural and “produced against nature.”

**Table 11:** Sydney Gen Z's perceptions about cultured meat being normal/not normal in the future.

	**Normal**	**Not normal**
1	“*Cultured meat is not natural for us to eat but in the future, this could be our only option to secure food. It could contain good nutrients as it is scientifically made, and everything is additionally added to make it taste better*.” (daily meat eater, community worker, age group 18–20 years)	“*It is absolutely not normal to consume cultured meat. It's out of my food comfort zone. I even don't want to try it*.” (daily meat eater, office assistant, age group 21–24 years)
2	“*Not sure if it is normal to eat now, but if no any other alternatives are available maybe people will start eating cultured meat*.” (daily meat eater, executive assistant, age group 21–24 years)	“*Meat substitutes are normal and quite popular even fashionable lately among young people like me. But lab meat is purely food-based biotech. Very abnormal for me and I will never eat these foods*.” (a few times per week meat eater, assistant, age group 21–24 years)
3	“*It is not normal for my diet right now to eat cultured meat, but with more information and with practice things could change and could become normal in the future*.” (daily meat eater, executive assistant, age group 21–24 years)	“*Meat alternatives are emerging and trendy now, but except some of my friends that are eating plant-based I don't think anyone who knows the taste of real steak to be willing to eat anything else including cultured meat although it is supposed to be a duplicate of a real meat*.” (a few times per week meat eater, retail team leader, age group 21–24 years)
4	“*The food of the future when there will be not enough food – if people like eating cultured meat I see no issue with the food shortages or with the future acceptance*.” (daily meat eater, netball coach, age group 18–20 years)	“*I think cultured meat is an unnatural nonsense*.” (occasional meat eater, shop assistant, age group 18–20 years)
5	“*Unfamiliar with cultured meat. I heard it's very expensive to produce and energy consuming. If cultured meat is made to exactly replicate meat in couple of years nobody will question from where the meat is coming from. People don't care much about it anyway*.” (a few times per week meat eater, administrative assistant, age group 21–24 years)	“*In vitro is completely unnatural thing. The meat gains its flavor from the animal, the amount of fat content, the marbling. It is a reflection of the way the animal is grown - the pasture it's grazed on, the food it's being fed. These all bring the flavor to the actual meat. While cultured meat is an artificially produced and flavored, not natural*.” (a few times per week meat eater, library services, age group 18–20 years)
6	“*Not now as they are not suitable for consumption, but I think cultured meat could become the new food social norm when it becomes more natural and accessible for humans to adopt it*.” (a few times per week meat eater, bookkeeper, age group 21–24 years)	“*I wouldn't eat stem cell based artificial meat from a lab. Animal stem cells, muscles are used to create a piece of meat. It's not normal. It's totally sick. It's really scary to think about, not even to consume it*.” (a few times per week meat eater, office assistant, age group 18–20 years)
7	“*Lab-Grown Meat as meat substitute will become normal if it is culturally accepted as now it is more looked as an artificial, engineering creation*.” (a few times per week meat eater, administrator, age group 21–24 years)	“*Chemically produced cell grown food can't be normal to consume. They can mimic the meat nutrition, but actually they are not as nutritious as meat. Marketers can say anything, but I am sure they serve someone's agenda*.” (few times a week meat eater, business development officer, age group 21–24 years)
8	“*Now cultured meat seems strange, but with the time when engineers improve the prototypes, it can look and taste and bleed like a real meat. Engineers almost done it with the plant-based meat*.” (a few times per week meat eater, case worker, age group 21–24 years)	“*I rather go vegetarian or vegan than eating cultured meat. It's not natural or normal…*.” (a few times per week meat eater, finance officer, age group 21–24 years)

#### Cultured Meat vs. Other Alternatives

When asked to express their opinion about different alternatives to livestock-based meat, namely edible insects, plant-based meat and cultured meat, according to their acceptance and preferences, the Gen Z participants were divided into five groups (see Table 12). One of the groups (*n* = 38, 17%) rejected all alternatives, including cultured meat. They were seen as “chemically produced,” “heavily processed,” and “not what our generation needs.” Another group (*n* = 25, 11%) rejected all alternatives in favor of increased consumption of fruit and vegetables: “I will stick to pure veggies” and “why… not eating normal veggies, we know they are good.” A larger group (*n* = 79, 35%) rejected cultured meat and edible insects but accepted plant-based alternatives because they “sound more natural” and are “normal.” Cultured meat was acceptable or possibly acceptable to the fourth group (*n* = 64, 28%) “if we can master it” as this will be “new forms of protein.” The fifth group (*n* = 21, 9%) accepted edible insects but rejected cultured meat because “it's too artificial and not a natural food like insects” and “innovations can try to change the meat industry but can't easily change the consumers who prefer natural stuff.”

**Table 12:** Cultured meat vs. alternatives according to Sydney Gen Z.

**Pro cultured meat**	**Pro plant-based alternatives, against cultured meat and edible insects**	**Pro insects, against cultured meat**	**Against all meat alternatives, including cultured meat**	**Against all new alternatives in favor of traditional vegetarian diet**
“*If we can master it and grow it out of bones, maybe cultured meat products will help us to change our attitude to animals and we can save the planet*.” (a few times per week meat eater, project coordinator, age group 21–24 years)	“…. *Except plant-based, the rest of the alternatives, cultured meat especially and the insects to some extent, are a bit odd food to eat… I am keen to follow the plant-based trend, but not the cultured meat*.” (daily meat eater, childcare educator, age group 21–24 years)	“*In the future, it will be necessary to eat edible insects because food and meat require lots of land to produce but I don't think that cultured meat will be something that people will like eating. It's too artificial and not a natural food like insects*.” (a few times per week meat eater, coordinator, age group 21–24 years)	“*Not considering in vitro or plant-based meats as natural as they are chemically produced to imitate meat*” (a few times per week meat eater, councilor, age group 21–24 years)	“*It is necessary as we are consuming vast quantities of meat and this is harmful for both the environment and our health, but honestly I wouldn't eat any clean meat or other plant-based as they are too processed. I prefer becoming a vegetarian, but not eating these meat mimicking ‘meats'*”. (a few times per week meat eater, teachers' aid, age group 21–24 years)
“*One day when we no longer have space to farm animals like cows it may be necessary to consume new meat alternatives and in vitro meat. We should start preparing ourselves as these lab meats could be our only chance*.” (a few times per week meat eater, IT analyst, age group 21–24 years)	“*Plant-based products to me sound more natural, the in vitro option does not seem natural*.” (daily meat eater, pharmacy assistant, age group 18–20 years)	“*You can't change our traditional diet with lab innovations. I rather eat bugs and other natural stuff than lab meat. Innovations can try to change the meat industry but can't easily change the consumers who prefer natural stuff*.” (daily meat eater, IT network, age group 21–24 years)	“*All plant- and lab-based meat alternatives are heavily processed to mimic meat. Maybe they are claimed good for the animals, for the environment, but how good lab meat could be if it is grown in an artificial way*?” (occasional meat eater, assistant, age group 18–20 years)	“*Why we should tolerate heavily processed stuff like lab meat and plant-based things to experience new food but not eating normal veggies, we know they are good? I don't get it*.” (a few times per week meat eater, roster coordinator, age group 18–20 years)
“*Investigating new forms of protein like lab meat are a good option and alternative for the growing humanity*.” (a few times per week meat eater, assistant operation manager, age group 21–24 years)	“*Plant based alternatives are normal, but the rest – cultured meat and the crickets and larvae are totally sick*” (daily meat eater, digital analyst, age group 21–24 years)	“*Cultured meat is not appealing, I feel it is the worst option even compared to crickets and other bugs we could have for our future food security*.” (non-meat eater, kitchen hand, age group 18–20 years)	“*Innovative solutions to replace meat with artificial meat and plant-based meats are not what our generation needs. We want clean, natural food*.” (occasional meat eater, university student, age group 18–20 years)	“*I wouldn't eat cultured meat, crickets and larvae. I can't put them into my mouth. I will stick to pure veggies*.” (occasional meat eater, human resources trainee, age group 21–24 years)

#### Embracing Meat Alternatives

**Figure 3:**
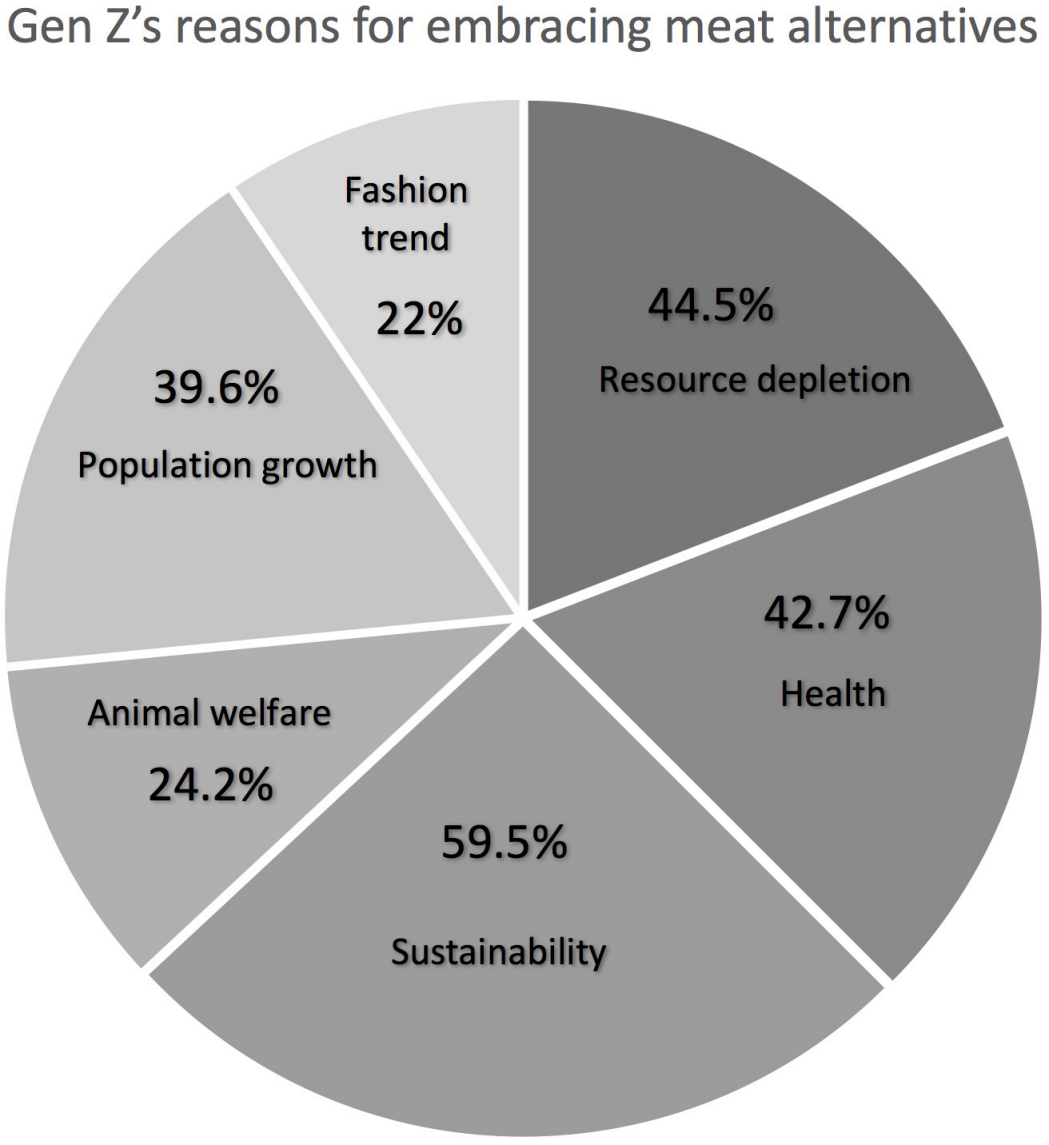
Reasons for embracing meat alternatives by Sydney Gen Z.

What could make Gen Z embrace alternatives to traditional animal meat, including cultured meat, is an important question related to the future of food. The question allowed multiple choices and Figure 3 presents the frequencies for each indicated answer. Broader sustainability concerns, including environmental impacts and contribution to climate change, was the most preferred answer (59%, *n* = 135), followed by specifically resource depletion (44%, *n* = 101). Other reasons are: health concerns (43%, *n* = 97), population growth (40%, *n* = 90), animal welfare (24%, *n* = 55), and fashion trend (22%, *n* = 50). These results confirm that Gen Z is concerned about the natural environment and is likely to adopt an eco-friendlier lifestyle (42).

## Discussion

Some of the findings from this exploratory study of adult Sydney Gen Z were expected and in line with previous research while others are unexpected. They all offer insights into this new generation and potential consumers of novel foods.

Sydney Gen Z's attitudes are very similar to the consumers' concerns in the West summarized by Bryant and Barnett (20) when it comes to the unnaturalness, safety, healthiness, anticipated taste and appearance of cultured meat. The residents of the largest Australian multicultural metropolis are used to a diversity of food options but the artificial and technology-based nature of cultured meat is making it difficult for the majority of them to accept this as a future choice in their diets. Some of their objections are grounded in the lack of sufficient information, but also in the almost instinctive feeling of disgust—a food-related emotion which plays a major role in building cognitive-affective linkages that guide behavior (43).

A 2020 study conducted in USA (44) shows a much higher acceptance of cultured meat amongst general consumers than what Sydney Gen Z indicates. Assuming that the price of cultured meat is the same as animal-based meat, 53% of the American consumers are prepared to make the substitution. Price considerations do not appear to be of any concern to Sydney's young adults but still only 28% of them consider cultured meat acceptable. Furthermore, 56% of the American consumers are prepared to substitute farmed meat for plant-based alternatives (44), while this percentage is lower at 35% for Sydney's Gen Z. These comparisons however should be treated with caution as the surveys conducted in the US and Australia use different descriptions of cultured meat, as well as different question formats, which can affect the responses.

Another study conducted in the USA shows that younger people in particular are more inclined to try cultured meat with 51% of those aged between 18 and 29 willing to do so (45). This share is again much lower at 28% amongst the Sydney Gen Z. A study in Germany (46) shows that children and adolescents are more willing to consume a burger made of cultured meat than of insects but it also confirmed the negative influence of neophobia. Similar attitudes were also manifested among the participants in our survey; however the feeling of disgust was quite pronounced in Australia whilst missing in Germany.

Cultural dimensions related to perceptions about masculinity and Australia's pride in producing high-quality animal meat products, are adding additional weight to the objections Gen Z has against alternatives to farmed meat. Although they do not explicitly express any considerations specifically about farmers (20, 47) or concerns about how farmed meat is produced (48), the way others have done previously, Gen Z seems to value Australia's reputation about being a supplier of quality livestock-based meat.

In 2019, many Gen Z students and young people actively participated in the climate strikes in Sydney raising their voices against greenhouse gas emissions, continuing deforestation and biodiversity loss. The issue about meat consumption was not part of the activists' agenda despite ample scientific evidence about the livestock's high environmental footprint. It seems that Gen Z has not been exposed enough to reliable information about farming practices in Australia, such as the use of antibiotics in poultry farms and the ecological footprint of the Australian beef. Only 41% express environmental concerns associated with the way meat is being currently produced. It is therefore important to educate young people about the environmental impact of farmed meat production.

If cultured meat is to replace livestock-based proteins, it will have to emotionally and intellectually appeal to the Gen Z consumers. It may be through its physical appearance, but what seems to be more important is transparency about its environmental and other benefits.

This generation has vast information at its fingertips, but is still concerned that they will be left with the legacy of exploitative capitalism that benefits only a few at the expense of many. They have witnessed such behavior resulting in climate change and are now afraid that a similar scenario may develop in relation to food, particularly as investors are pursuing broader adoption (48). The conspiracy theory identified as a major concern in relation to edible insects (29), also rings alarm bells in the case of cultured meat. Conspiratorial ideation related to rejection of cultured meat was also identified in the study by Wilks et al. (49).

With the exception of 19% (*n* = 43), the remaining participants did not share proper conceptual knowledge about the way cultured meat is made. As explained by Madden (30, p. 193), this characteristic is a result of Gen Z being “brokers of information rather than knowers of content.” They value the opportunity to access current information when needed, but not memorizing the content for future use. In this sense, it is likely that when cultured meat becomes available at the Australian market, Gen Z will start looking for answers in order to understand the “why” behind the “what” (30).

Devoted to their true technological nature some of the participants find fascinating the technological advancement of humanity to create *in-vitro* meat. They also see this as an opportunity to prevent further animal suffering and reduce the environmental impacts of livestock production. This generation appears to be opening the door to the miracles of technology for the achievement of broader societal benefits with 28% (*n* = 64) already accepting cultured meat. Focussing on the benefits of the final product, not the way it is produced (50), may increase acceptance.

Shaped with increased global awareness, Gen Z is particularly cautious about the impact of their product choices. What matters the most is the ethical side of production (30) and this was demonstrated through their concerns about animal dignity in the process of cultured meat. If the produced outcomes are environmentally and ethically good, it will be worth considering.

The participants indicated that they do not have a “fear of missing out” (30) when it comes to cultured meat. They do not prioritize concerns about establishing competitive advantages, the anticipated price of cultured meat or whether the process will be properly regulated and controlled. Living in a prosperous and stable economic environment with ample food availability, Sydney Gen Z values higher its freedom of choice, be it to opt for the traditional fruit and vegetables, than being the early innovators.

These young adults have also been exposed to the Covid-19 pandemic which is challenging human relationships with food from different angles (51). Large sections of natural habitat have been destroyed for the purpose of producing meat and growing animal feed, threatening further the survival chances of thousands of species. As our respondents indicated, many people are likely to seriously consider the environmental impacts of their food choices and opt for better options. Ironically, during the spread of the pandemic many abattoirs, meat and poultry plants in Australia and around the world became clusters for coronavirus cases (52). Cultured meat may soon be perceived as safer and not posing health risks, because it is produced in a virus-free sterile environment where no antibiotics are used. When faced with such choices, people may soon find the cultured meat decision more attractive.

While the future of cultured meat is still hanging in the balance, Gen Z consumers are similarly in the waiting. They are also open to be convinced. Being an exploratory qualitative study, it provides new insights about a demographic population that has not been studied previously and opens up opportunities for further statistically significant explorations.

## Conclusion

According to Stephens et al. (7), whether cultured meat would really become clean, ethical and with a low environmental impact will depend on the efficiency of the production processes and the motivations of the companies and their funders. For the time being, Gen Z is not ready to embrace cultured meat with 72% finding it not acceptable. Emotional feelings of disgust dominate their individual attitudes toward the anticipated taste of cultured meat. Many are prepared to opt for plant-based meat alternatives and even the traditional fruit and vegetables instead. Australia's Gen Z is not concerned about the price, regulations or controls surrounding cultured meat, however some doubt the motivation behind investing in these technological developments. A major concern for Gen Z are the healthiness of cultured meat and its unnaturalness with 32% being of the opinion that it is not a healthy option.

Nevertheless, 28% of Gen Z are prepared to try cultured meat and some (25%) find it an option which can help with population growth. As 41% already acknowledge the need to switch to more sustainable food choices, further information related to the environmental implications, animal welfare and health impacts of farming livestock may sway these young people's opinion. Any persuasion however should be logical, with a clear purpose and no hidden messages as Gen Z are independent thinkers, conscientious about their food choices and prefer to find the answers themselves. Being digitally savvy and highly connected, they will also have to address the challenges the previous generations have thrown at them, including climate change, environmental health and new emerging zoonotic diseases.

It is not surprising that Gen Z's value system is penetrating its food choices. As each generation makes its mark on the world, it may well be that Gen Z is the one putting cultured meat to the test and deciding its future.

## Data Availability Statement

The raw data supporting the conclusions of this article will be made available by the authors on request.

## Ethics Statement

The studies involving human participants were reviewed and approved by Curtin University. The patients/participants provided their written informed consent to participate in this study.

## Author Contributions

DB and DM conceptualized the study, analyzed the data, and contributed equally to all the sections in the article. DB conducted the survey. All authors made substantial contributions throughout all sections, read, and approved the final manuscript for publication.

## Conflict of Interest

The authors declare that the research was conducted in the absence of any commercial or financial relationships that could be construed as a potential conflict of interest.
